# Optimised PCR assays for detecting elusive waterfowl from environmental DNA

**DOI:** 10.1002/ece3.11224

**Published:** 2024-04-01

**Authors:** Johanna Honka, Laura Kvist, Suvi Olli, Toni Laaksonen, Jouni Aspi

**Affiliations:** ^1^ Ecology and Genetics Research Unit University of Oulu Oulu Finland; ^2^ Department of Biology University of Turku Turku Finland; ^3^ Natural Resources Institute Finland (Luke) Helsinki Finland

**Keywords:** *Anser fabalis*, bean goose, *Branta*, *Cygnus*, eDNA, mitochondrial DNA

## Abstract

For many aquatic and semiaquatic mammal, amphibian and fish species, environmental DNA (eDNA) methods are employed to detect species distribution and to monitor their presence, but eDNA is much less employed for avian species. Here, we developed primers for the detection of true geese and swan species using eDNA and optimised a PCR protocol for eDNA. We selected taiga bean goose (*Anser fabalis fabalis*) as our focal (sub)species and sampled water from lakes, from which the presence of taiga bean goose was visually confirmed. To test, if taiga bean goose DNA could be detected among DNA of other goose species, we similarly sampled eDNA from a zoo pond housing several Anatidae species. We were able to detect taiga bean goose DNA in all but one of the tested lakes, including the zoo pond. The primers developed are not species‐specific, but rather specific to the genus *Anser*, due to the close relatedness of *Anser* species, which prevented the development of species‐specific primers and the use of, for example, quantitative PCR. We also developed eDNA primers for *Branta* species and *Cygnus* species and tested these primers using the same samples. Canada goose (*B. canadensis*) and barnacle goose (*B. leucopsis*) DNA were only detected in the zoo pond (in which they were present), as the sampled natural lakes fall outside the range of these species. We detected whooper swan (*C. cygnus*) DNA in three lakes and the zoo pond (in which the species was present). The eDNA method presented here provides a potential means to monitor elusive goose species and to study the co‐occurrence of large waterfowl.

## Graphical abstract

We developed primers for the detection of true geese and swan species using environmental DNA (eDNA) and optimised a PCR protocol for eDNA. We show that eDNA is effective at detecting geese and swans without the need to disturb the animals, providing an alternative method for determining occupancy and distribution of large waterfowl.

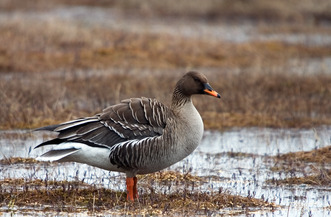

## 1 INTRODUCTION

Within recent decades, environmental DNA (eDNA), that is DNA extracted from the environment such as water, soil, snow, or air (Taberlet, Coissac, Hajibabaei, & Rieseberg, 2012), has been successfully employed to detect and monitor various aquatic and terrestrial animal species (Barnes & Turner, 2016; Beng & Corlett, 2020). Despite the usefulness of eDNA in detecting and monitoring animal species, eDNA applications in birds are still limited. In zoo environments, studies have demonstrated that avian eDNA can be sampled from pond water (Ushio et al., 2018) and from air (Clare et al., 2022; Lynggaard et al., 2022). The existing eDNA studies of wild bird species include determining the distribution of the North American marshland bird, the black rail (*Laterallus jamaicensis*) (Feist et al., 2022; Neice & McRae, 2021) and the Ridgway's rail (*Rallus obsoletus*) (Guan et al., 2023), detecting an endangered land bird, the Gouldian finch (*Chloebia gouldiae*), from drinking sites (Day et al., 2019), as well as detecting the kākāpō (*Strigops habroptilus*) from soil samples (Urban et al., 2023). Furthermore, studies have focused on detecting various avian species in diverse habitats, including urban waterbodies (Zhang et al., 2023), natural wetlands (Coleman et al., 2023), leaf swaps (Lynggaard et al., 2023), airborne dust (Johnson et al., 2023), air (Lynggaard et al., 2024), and spider webs (Newton et al., 2024). Additionally, several bird pollinators and an insectivorous bird were identified from eDNA collected from flowers (Jønsson et al., 2023; Newton et al., 2023). eDNA primers have been developed for some wading birds (*Platalea leucorodia*, *Recruvirostra avosetta* and *Tringa tetanus*), but these have not been tested in the wild (Schütz et al., 2020). From ancient eDNA, for example, an ancient *Branta* goose has been identified from sediment samples approximately 2 million years old employing a metagenomic approach (Kjær et al., 2022).

Monitoring of avian species is crucial as close to half of the world's bird species are declining, and about 1500 bird species (14% of all avian species) are facing a global extinction risk (Lees et al., 2022). Climate change poses a significant threat, particularly to northern species at risk of losing their habitats. Water bodies, such as lakes, ponds, rivers, or oceans could be highly useful sources of eDNA for monitoring or detecting the presence of aquatic or semiaquatic bird species, especially those that visit water bodies regularly, such as waterfowl (Anseriformes). Detection and monitoring of avian species using eDNA have the benefits that birds do not need to be captured or disturbed for sampling or even sighted or heard, which could greatly improve the monitoring of rare and/or elusive species. Furthermore, collecting water samples for eDNA does not require specialised expertise or extensive bird identification skills that necessitate lengthy training. It could thus be performed by non‐experts, for example, as citizen‐science projects. Traditional bird breeding mappings may require several years and intensive fieldwork to complete, and eDNA methods could significantly expedite distribution mapping for waterfowl.

**FIGURE 1:**
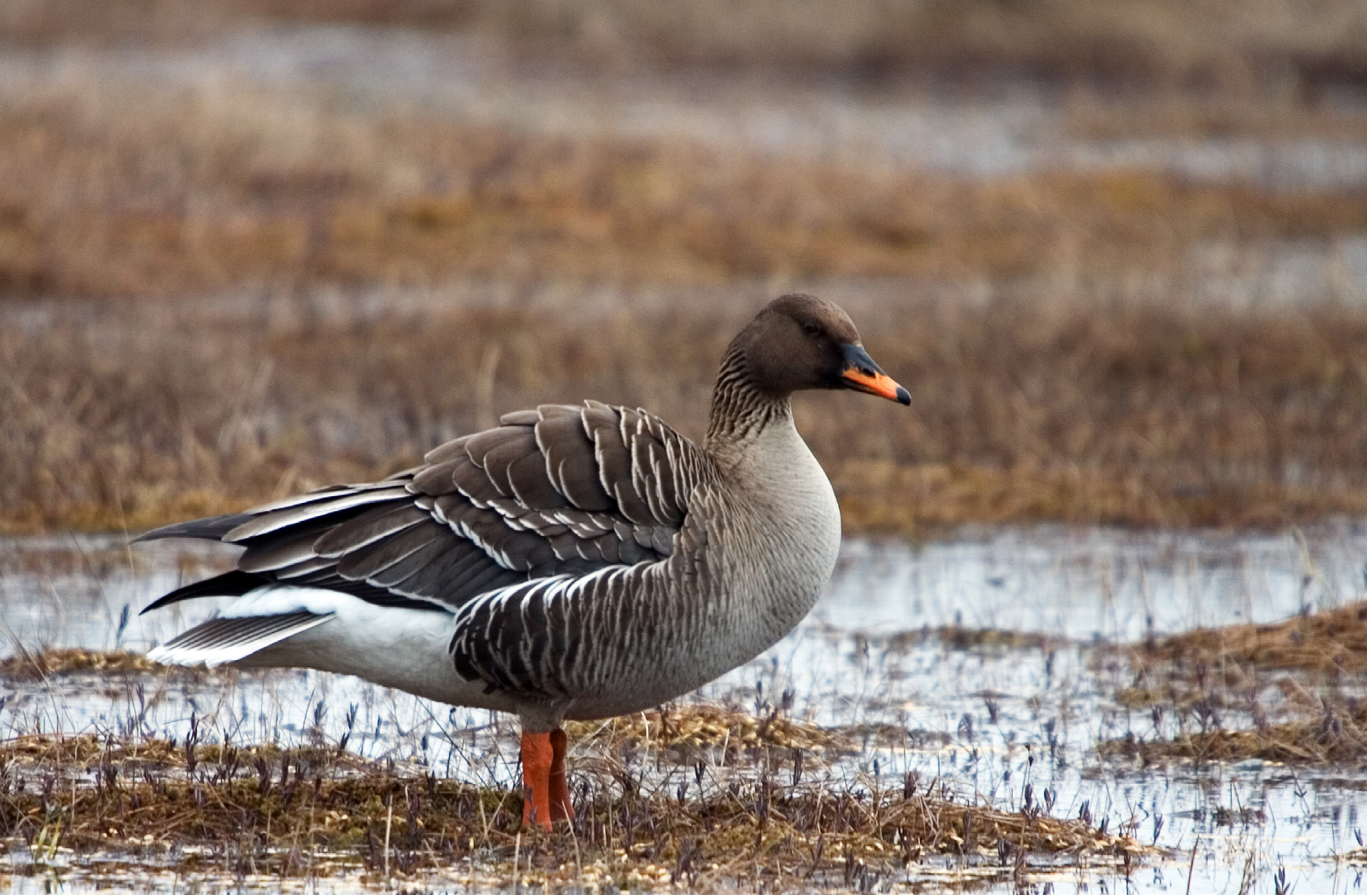
One of the target species, semiaquatic taiga bean goose (*Anser fabalis fabalis*) on a mire. Photo: Seppo Kemppainen.

Many goose species are highly elusive during the breeding season, making them notoriously difficult to monitor. An eDNA‐based detection method would greatly enhance the detection of these shy species. While most goose populations have increased in numbers in recent decades, certain populations have declined and are of conservation concern, such as the taiga bean goose (*Anser fabalis fabalis*; Figure 1), and some populations are critically endangered such as the Fennoscandian lesser white‐fronted goose (*A. erythropus*). Others, such as the tundra bean goose in Finland (*A. f. rossicus*), are endangered with poorly known distribution. Certain populations are of management concern due to conflicts with humans and agriculture as their numbers are in increase (greylag goose, *A. anser*; pink‐footed goose, *A. brachyrhynchus* and barnacle goose, *B. leucopsis*), or due to tundra vegetation degradation (pink‐footed goose), or introductions to non‐native locations (Canada goose, *B. canadensis*).

There is a particular need to develop new monitoring methods for elusive declining species that breed in remote and difficult‐to‐access Arctic areas (Johnson et al., 2021; Markkola, 2022; Pirkola & Kalinainen, 1984). For example, the population of the most endangered bird in Europe, the lesser white‐fronted goose (currently numbering 25–30 breeding pairs) has increased in numbers, but the breeding locations of all individuals remain unknown. An eDNA‐based method could be used to map the former breeding range of this species, verify if there are re‐colonised nesting locations and target protection to these sites. This species is elusive during breeding and field observations could consume numerous hours of work in the field with no road network, making an eDNA‐based method timesaving.

It has been proposed that the increased population of whooper swans (*Cygnus cygnus*) has contributed to the decline of the taiga bean goose population due to the aggressiveness of whooper swans toward bean geese (Kampe‐Persson et al., 2005). Using an eDNA‐based detection, it would be possible to study the co‐occurrence of these species during breeding time. While the whooper swans are relatively easy to detect due to their white plumage and large size, the highly elusive bean geese could be easily overlooked if inhabiting the same lake. Further, by adapting the eDNA method to lake bottom sediment samples (sedimentary ancient DNA, sedaDNA), the historical presence and distribution of waterfowl species could be determined, providing information about the presence of the target waterfowl in the past, and at the same time, also possibly about the past environmental conditions. However, eDNA assays are currently lacking for any waterfowl species.

Due to a higher copy number of mitochondrial DNA (mtDNA) and its simple maternal inheritance, mtDNA is usually the marker of choice for eDNA assays. The true geese (*Anser* and *Branta*) are closely related, particularly within the genus *Anser* (Ruokonen et al., 2000), and have experienced high levels of ancient hybridisation events and gene flow (Ottenburghs et al., 2017). Several *Anser* species exhibit a highly similar DNA barcoding (*COI*) region (Johnsen et al., 2010), making this region unsuitable for primer development. Even in the most variable region of mitochondrial DNA, the control region, the differentiation between *Anser* species is low (0.9%–5.5%) (Ruokonen et al., 2000).

**FIGURE A1:**
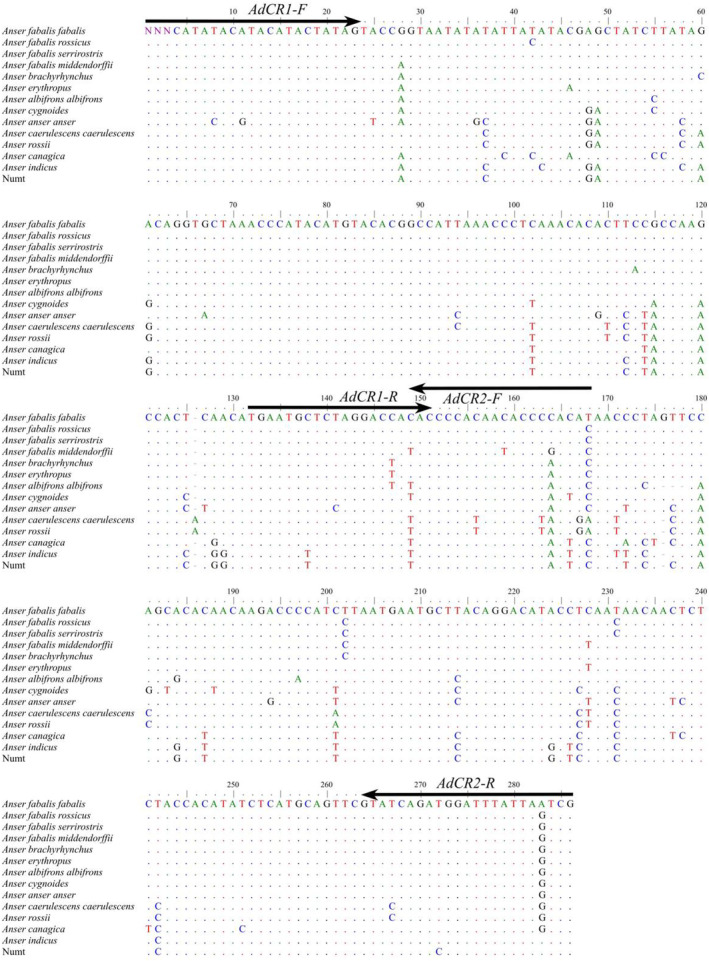
Sequence alignment of a part of a control region for *Anser* geese species with one sequence per species, except for bean goose subspecies (GenBank accession numbers: EU186807, EU186812, EU186810, EU186805, AF159952, AF159955, AF159957, AF159961, AY072581, FJ905228, AY072582, AY072583 and KM455570), in addition to Numt (nuclear sequence of mitochondrial origin; GenBank: AF159970). Locations of tested primers are shown as arrows. The primer pair selected for environmental DNA analyses was AdCR2‐F/R.

Additionally, mitochondrial DNA can be also found copied in the nuclear DNA, known as a Numt (nuclear sequence of mitochondrial origin; Lopez et al., 1994). This is the case also with *Anser* geese (Ruokonen et al., 2000). Due to close relatedness, sequence similarity and the presence of Numt sequences, the development of species‐specific primers even for the most variable mtDNA control region is not possible for true geese (see Honka et al., 2018 and Figure A1 in Appendix).

Quantitative PCR (qPCR) and digital droplet PCR (ddPCR) are sensitive methods to detect single species from eDNA samples, but these would require highly species‐specific primers. Due to a lack of a DNA region with enough sequence variation between species, we opted for Sanger sequencing of the PCR‐amplified amplicons to verify the target species. The *Anser* primers used here were developed to contain mismatches to Numts (Honka et al., 2018, see Figure A1 in Appendix).

**FIGURE A2:**
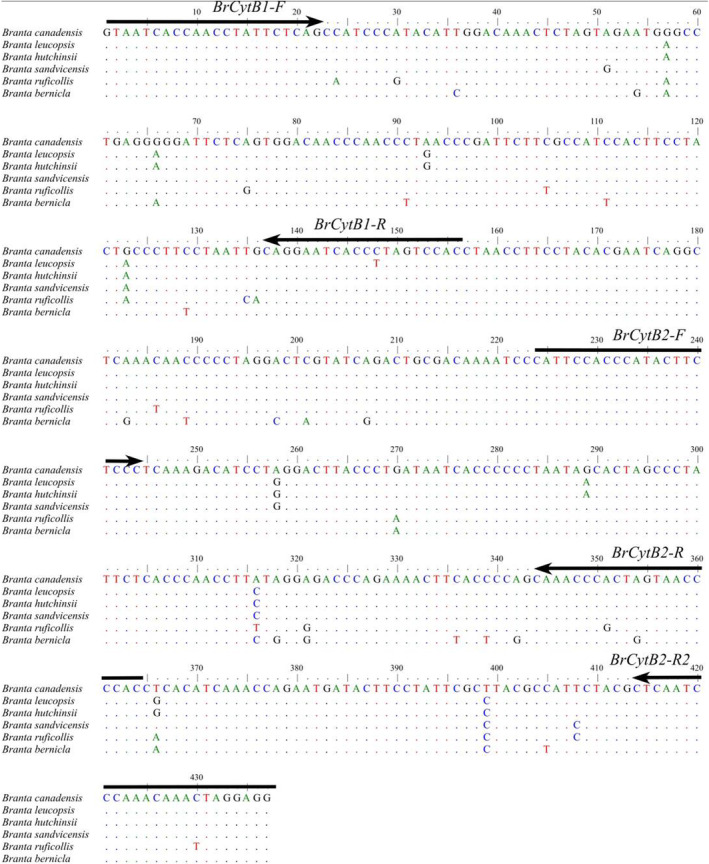
Sequence alignment of a part of a *Cytochrome B* gene for *Branta* geese species with one sequence per species (GenBank accession numbers: EU585629, EU585630, MH676095, EU585632, EU585631, and EU585628). Locations of tested primers are shown as arrows. The primer pair selected for environmental DNA analyses was BrCytB2‐F/R2.

**FIGURE A3:**
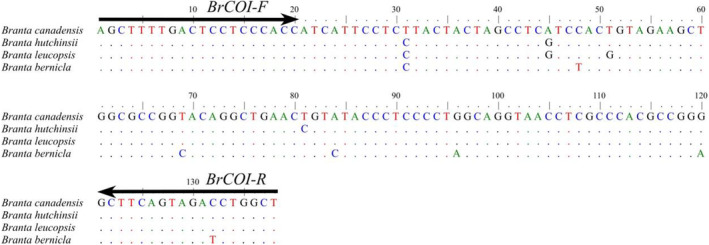
Sequence alignment of a part of a *Cytochrome oxidase I* (*COI*) gene for *Branta* geese species with one sequence per species (GenBank accession numbers: NC_007011, MH676092, GU179003 and KJ680301). Hawaiian goose (*B. sandviciensis*) and red‐breasted goose (*B. ruficollis*) lacked *COI* sequence from the GenBank. Locations of tested primers are shown as arrows. The primer pair was not used for environmental DNA.

**FIGURE A4:**
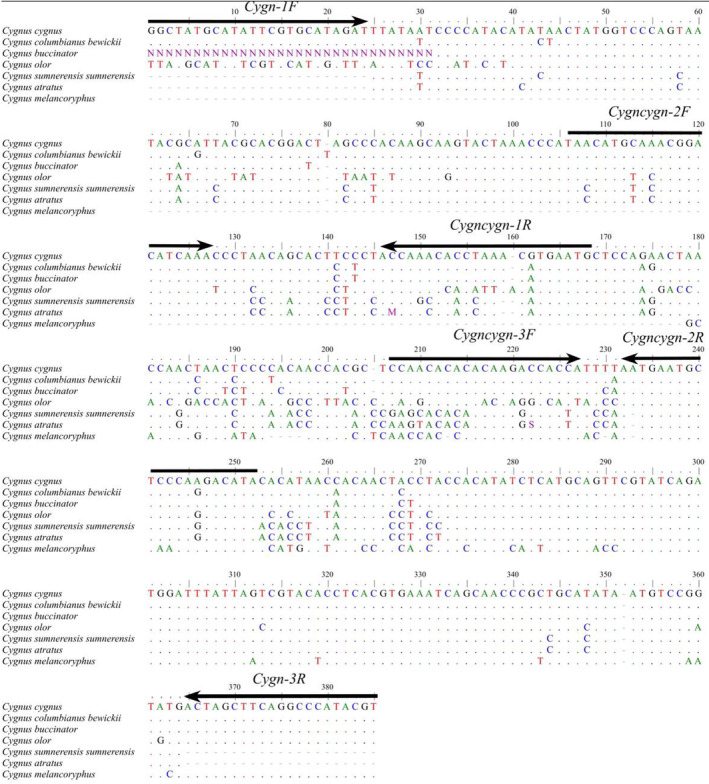
Sequence alignment of a part of a control region for *Cygnus* species with one sequence per species (GenBank accession numbers: NC_027095, NC_017604, EF165358, NC_027096, MF455379, MF455395 and KY463444). This alignment also contains a sequence of an extinct New Zealand swan (*C. sumnerensis*) for which the primers were originally developed for but modified in this study. In addition, this alignment includes black‐necked swan (*C. melancoryphus*) as a partial sequence. Locations of tested primers are shown as arrows. The primer pair selected for environmental DNA analyses was Cygn‐1F and Cygngygn‐1R.

Similarly to *Anser*, species‐specific primers were found to be impossible to develop for *Branta* species, with the added difficulty that some primers co‐amplify *Anser* and *Branta* species (this study, see below Section 2; Figures A2 and A3 in Appendix). Furthermore, the Northern swan species whooper swan (*C. cygnus*) and tundra swan (*C. columbianus*) have high sequence similarity within the barcoding (*COI*) region (Johnsen et al., 2010), and thus control region was used for swans as well. The *Cygnys* primers (modified from Rawlence et al., 2017) were also found not to be species‐specific based on sequence alignment (Figure A4 in Appendix), and we also opted for Sanger sequencing. Because the primers used here are not species‐specific, it is crucial to verify the species by sequencing the amplicons.

The purpose of this study was to develop and optimise eDNA‐based assays for elusive waterfowl. We (1) tested several primer pairs that amplify a short region of mitochondrial DNA from each genus to select the best‐performing primer pair for analyses of eDNA. In addition, (2) we optimised PCR protocols for eDNA samples by comparing different protocols and polymerases and Sanger sequenced the amplicons to identify the species. We further (3) discuss our results for usability in monitoring endangered or invasive populations and mapping their breeding distribution. In this study, we pilot the use of eDNA methods in waterfowl, for which markers suitable for eDNA have not been developed or tested before, focusing on large waterfowl in the Northern Hemisphere: the true geese (*Anser* and *Branta*) and the swans (*Cygnus*).

## 2 MATERIALS AND METHODS

### 2.1 Study sites and water sampling

We collected water samples from natural lakes and a zoo pond from northern Finland and extracted eDNA from the samples. In all water bodies, from which samples were taken, a visual observation of taiga bean goose (Figure 1) was confirmed allowing us to test for (1) the presence of taiga bean goose DNA in all samples. We also adopted this for the (2) Canada goose and barnacle goose, only present in the zoo pond and for (3) the whooper swan (*C. cygnus*) which was visually observed in one of the natural lakes and the zoo pond but breeds throughout Finland and is thus potentially present in any of the lakes. As *Branta* species were not visually detected in the natural lakes and have more southerly ranges, we were able to check for false positives in our eDNA. Water samples were retrieved in triplicates (3 samples per lake) from six natural lakes in Finland during the period of 17th of July to 16th of August 2018 (Table 1), based on confirmed sightings of bean geese. Water samples were filtered either on‐site or collected in sterile 50 mL Falcon tubes and filtered within a few hours (Table 1). Lake water was collected using a sterile 50‐mL syringe with a Luer‐Lok tip (VWR) either directly from lake water or the Falcon tubes and filtered using a Sterivex‐GP Pressure Filter Units with 0.22 μm pore size and Male Luer‐Lok inlet (Merck/Millipore).

**TABLE 1:** Sequencing results from the environmental DNA samples collected from various lakes and one zoo pond targeting for the genuses *Anser*, *Branta* and *Cygnus*.

Sample name	Sampling site	Sampling date	Filtration	Amount of sampled water (mL)	Visual observation	Species based on primers AdCR2‐F and AdCR2‐R	Species based on primers BrCytB2‐F and BrCytB2‐R2	Species based on primers Cygn‐1F and Cygncygn‐1R
EB1	Eastern Lapland	17/07/2018	On‐site	150–200	Taiga bean goose	Taiga bean goose^a^	N/a	N/a
EB2	Eastern Lapland	17/07/2018	On‐site	150–200	Taiga bean goose	Taiga bean goose^a^	N/a	N/a
EB3	Eastern Lapland	17/07/2018	On‐site	150–200	Taiga bean goose	Taiga bean goose^a^	N/a	N/a
EB4	Northern Lapland	22/07/2018	Water collected to sterile tubes	150	Taiga bean goose	N/a	N/a	N/a
EB5	Northern Lapland	22/07/2018	Water collected to sterile tubes	150	Taiga bean goose	N/a	N/a	N/a
EB6	Northern Lapland	22/07/2018	Water collected to sterile tubes	150	Taiga bean goose	N/a	N/a	N/a
EB7	Fjeld Lapland	22/07/2018	Water collected to sterile tubes	150	Taiga bean goose	N/a	N/a	N/a
EB8	Fjeld Lapland	22/07/2018	Water collected to sterile tubes	150	Taiga bean goose	N/a	N/a	N/a
EB9	Fjeld Lapland	22/07/2018	Water collected to sterile tubes	150	Taiga bean goose	Taiga bean goose^a^	N/a	N/a
EB10	Kemi‐Tornio region	23/07/2018	Water collected to sterile tubes	100	Taiga bean goose	Taiga bean goose^a^	N/a	N/a
EB11	Kemi‐Tornio region	23/07/2018	Water collected to sterile tubes	≈70	Taiga bean goose	Taiga bean goose^a^	N/a	Whooper swan
EB12	Kemi‐Tornio region	23/07/2018	Water collected to sterile tubes	50	Taiga bean goose	Taiga bean goose	N/a	N/a
EB13	North of Oulu region	02/08/2018	On‐site	100	Taiga bean goose, whooper swan	Taiga bean goose	N/a	Whooper swan
EB14	North of Oulu region	02/08/2018	On‐site	100	Taiga bean goose, whooper swan	Taiga bean goose	N/a	Whooper swan
EB15	North of Oulu region	02/08/2018	On‐site	100	Taiga bean goose, whooper swan	Taiga bean goose^a^	N/a	N/a
EB16	Oulu region	16/08/2018	On‐site	100	Taiga bean goose	N/a	N/a	N/a
EB17	Oulu region	16/08/2018	On‐site	100	Taiga bean goose	N/a	N/a	N/a
EB18	Oulu region	16/08/2018	On‐site	100	Taiga bean goose	Taiga bean goose and pink‐footed goose^c^	N/a	N/a
EB19	Ranua Wildlife Park	08/10/2018	On‐site	400	Taiga bean goose, greylag goose, lesser white‐fronted goose, barnacle goose, Canada goose, hybrid geese, whooper swan	Bean goose and greylag goose	Barnacle goose^b^ and Canada goose	Low‐quality whooper swan sequence
EB20	Ranua Wildlife Park	08/10/2018	On‐site	400	Taiga bean goose, greylag goose, lesser white‐fronted goose, barnacle goose, Canada goose, hybrid geese, whooper swan	Bean goose and greylag goose	Barnacle goose^b^ and Canada goose	Low‐quality whooper swan sequence
EB21	Ranua Wildlife Park	08/10/2018	On‐site	400	Taiga bean goose, greylag goose, lesser white‐fronted goose, barnacle goose, Canada goose, hybrid geese, whooper swan	Bean goose and greylag goose	Barnacle goose^b^ and Canada goose	Low‐quality whooper swan sequence

*Note*: Each site was sampled in triplicates from different parts of the lake or pond. To safeguard the breeding sites of the taiga bean goose (*A. fabalis fabalis*) specific lake names are withheld. Two distinct sampling methods were employed: directly from the lake (on‐site filtration) or water collected to sterile tubes. The amount of water filtered is also reported. Visual observation denotes instances in which the species was detected in or around the lake site a day or a few days before sampling or during the sampling process. Species identification was performed with Sanger‐sequencing using primer pair AdCR2‐F and AdCR2‐R (taiga bean goose; pink‐footed goose, *A. brachyrhynchus*; greylag goose (*A*. *anser*), primer pair BrCytB2‐F and BrCytB2‐R2 (Canada goose, *B. canadensis*; barnacle goose, *B. leucopsis*) or primer pair Cygn‐1F and Cygncygn‐1R (whooper swan; *C. cygnus*)). N/a indicates that no PCR band of the correct size was detected in an agarose gel.

^a^ The sample was excised and extracted from an agarose gel (multiple bands in gel).

^b^ Sequence identical with cackling goose (*B. hutchinsii*), but cackling goose does not breed in Finland, nor was housed in the zoo.

^c^ *A. brachyrhynchus* haplotypes have been also found from the taiga bean goose (Honka et al., 2022), thus the presence of the pink‐footed goose is uncertain.

Water was injected into the Sterivex Filter Unit until the filter became clogged, resulting in filtering volumes ranging from 50 to 200 mL depending on water turbidity. The filter cartridge was then emptied of water by injecting air with the syringe, and the filter outlet was sealed with a small piece of mouldable silicone ear plug (obtained from a pharmacy). We injected 2.5 mL of absolute ethanol into the filter cartridge using a sterile 3‐mL syringe with a Luer‐Lok tip (VWR) to preserve the filter (Spens et al., 2017). The filter inlet was capped with a Cole‐Parmer Animal Free Male Luer Lock Plug (Cole‐Parmer). Both the inlet and outlet were sealed with parafilm to ensure that the plugs did not open during the transportation, and the Filter Unit was placed in a Minigrip bag. The samples were stored at 4°C for 3–9 days before DNA extraction.

In addition to natural lakes, we similarly filtered three triplicate eDNA samples from a zoo pond in Ranua Wildlife Park on 8 October 2018 (Table 1). The zoo pond was inhabited by multiple Anatidae species: bean goose (*A. fabalis*; *n* = 5), greylag goose (*A. anser*; *n* = 3), lesser white‐fronted goose (*A. erythropus*; *n* = 3), Canada goose (*B. canadensis*; *n* = 1), barnacle goose (*B. leucopsis*; *n* = 3), two hybrid goose individuals (*B. leucopsis* × *B. canadensis* × *A. caerulescens* and *B. leucopsis* × *B. ruficollis*), whooper swan (*C. cygnus*), mallards and domestic ducks (*Anas platyrhynchos*). The pond is routinely emptied and washed every 2 weeks. The water sampling and filtering procedures were the same as those used for the natural lakes, except that 400 mL of water was filtered in each replicate.

### 2.2 Authentication of eDNA

All equipment were either sterile or pre‐washed with 10% bleach and rinsed with sterile water and 70% ethanol. All liquids taken to the field were aliquoted into sterile Falcon tubes within a laboratory room dedicated to samples with low amounts of DNA and in which no PCR products were handled. The laboratory room was UV‐treated prior to any work. The equipment was packed in Minigrip bags to reduce the chance of environmental contamination during the field sampling. We used single‐use gloves when taking the water samples and the gloves were changed between each replicate sample. To ensure that the equipment was not contaminated during the transportation to the field or during the field sampling, 100 mL of sterile water was transported to the field as field negative controls. The water was filtered and the filters were preserved similarly to the lake samples. Three field negative controls were taken: at the beginning of the sampling season (17 July 2018), in the middle of the sampling season (23 July 2018) and at the end of the sampling season (16 August 2018).

### 2.3 DNA extraction

All DNA extractions were also performed in the laboratory room dedicated to samples with low amounts of DNA and which was UV‐treated prior to any work. Only sterile filter tips were used in pipettes. All working surfaces and racks were wiped with 10% bleach and 70% ethanol. The outsides of the filter cartridges were also wiped with 10% bleach prior to DNA extraction.

To start the DNA extraction, ethanol within the filter cartridge was dispelled to 1.5 mL microcentrifuge tubes using a sterile 3 mL syringe. We utilised an ‘Open Sterivex’ method, in which the filter is separated from its casing (Cruaud et al., 2017). This procedure, compared to DNA extraction within the filter casing, does not require specialised equipment and has been shown to increase DNA yield (Cruaud et al., 2017). The filter cartridge was cut open from the outlet end using flame‐sterilised PVC pipe cutters. After each sample, the cutters were washed with deionised water, dried with tissue paper, and flame‐sterilised. The filter was then cut into small pieces on a petri dish using a sterile disposable scalpel following the instructions in Cruaud et al. (2017). Any remaining ethanol was allowed to evaporate, and the filter pieces were transferred to a 2 mL screw cap microcentrifuge tube using flame‐sterilised forceps. The field negative controls were processed similarly.

DNA was extracted using the DNeasy Blood and Tissue Kit (Qiagen) following the tissue protocol, except for adding 720 μL of ATL buffer and 80 μL of proteinase K as described in Spens et al. (2017). Samples were incubated on a shaking heat block overnight (>20 h) at 56°C and 600 rpm. The amount of AL buffer was adjusted based on the sample volume and an equal amount of ice‐cold ethanol was added following Spens et al. (2017). We added 650 μL of the mixture in the Dneasy Mini spin column and repeated this until all the mixture was filtered through the spin column. DNA was eluted with 50 μL of AE buffer, the column was incubated at room temperature for 5 min, centrifuged, and the elution step was repeated. In addition to the field negative controls, we also processed DNA extraction negatives (no filter; *n* = 3) to control that the extraction kit and the equipment used were not contaminated. Extracted DNA was stored at −20°C.

### 2.4 Optimisation of the annealing temperature of the primers

We selected several primer pairs for each genus, *Anser*, *Branta*, or *Cygnus*, either based on literature (*Anser* and *Cygnus*) or developed for this study (Table 2, Figures A1, A2, A3, A4 in Appendix). Primers were manually developed by visually comparing multiple sequences of different species and identifying priming sites, which would flank variable regions with nucleotide differences between different species. We aligned the mtDNA control region sequences as well as several mtDNA gene sequences: *COI*, *Cytochrome B*, and *NADH2*. The melting temperatures were calculated to be within <5°C between the forward and the reverse primers. We also ensured that there was no self‐priming within the primers with the OligoCalc tool (Kibbe, 2007). Additionally, we attempted to identify sites for species‐specific primers, but the sequences did not have enough variation to design such primers.

**TABLE 2:** Primers suitable for environmental DNA (eDNA) for the genera *Anser*, *Branta* and *Cygnus*, with annealing temperatures (°C), PCR product sizes (bp, base pair), targeted mitochondrial gene or region (COI, *Cytochrome c oxidase*; CytB, *Cytochrome b*) and reference of the primers. In addition, primers tested to amplify the species, but not suitable for eDNA are also shown.

Genus	Primer	Primer sequence 5′–3′	Annealing temperature (°C)	Product size (bp)	Mitochondrial gene/region	References
Primers used for eDNA						
*Anser*	AdCR2‐F	TGAATGCTCTAGGACCACAC	57	111	Control region	Honka et al. (2018)
	AdCR2‐R	CGACTAATAAATCCATCTGATAC				
*Branta*	BrCytB2‐F	CATTCCACCCATACTTCTCCC	63	168	*CytB*	This study
	BrCytB2‐R2	CTCCTAGTTTGTTTGGGATTGAGC				
*Cygnus*	Cygn‐1F	GGTTATGCATATTCGTGCATAGAT	57	120	Control region	Butkauskas et al. (2012)
	Cygncygn‐1R	CATTCACGTTAGGTGTTTGGT				Modified from Rawlence et al. (2017) for this study
Primers tested that are suitable for tissue samples but not for eDNA						
*Anser*	AdCR1‐F	CCCCATACACGTACATACTATAG	57	123	Control region	Honka et al. (2018)
	AdCR1‐R	GTTGGGTGTTGTGGGGTG				
*Branta* ^a^	BrCOI‐F	AGCTTTTGACTCCTCCCACC	63	100	*COI*	This study
	BrCOI‐R	AGCCAGGTCTACTGAAGC				
*Branta* ^a^	BrCytB1‐F	GTAATCACCAACCTATTCTCAGC	63	115	*CytB*	This study
	BrCytB1‐R	GTGGACTAGGGTGATTCCTG				
*Branta* ^b^	BrCytB2‐R	GTGGGGTTACYAGYGGGTTTG	63	98	*CytB*	This study
*Cygnus*	Cygncygn‐2F	TAACATGCAAACGGACATCAAA	57	101	Control region	Modified from Rawlence et al. (2017) for this study
	Cygncygn‐2R	TATGTCTTGGGAGCATTCATT				
*Cygnus*	Cygncygn‐3F	CCAACACACACAAGACCACCA	57	138	Control region	Modified from Rawlence et al. (2017) for this study
	Cygn‐3R	ACGTATGGGCCTGAAGCTAGT				Rawlence et al. (2017)

^a^ Primers co‐amplify *Anser* and *Branta* species in environmental DNA samples.

^b^ Primer BrCytB2‐F was used as the forward primer. This PCR product was successfully sequenced only in one direction leaving with only 80 bp of sequence.

The primers for the genus *Cygnus* were originally developed for ancient DNA of black swans (*C. atratus*; Rawlence et al., 2017) and were manually modified (checking visually) in this study to match the *C. cygnus* GenBank sequence (GenBank accession number: JQ693392.1; Butkauskas et al., 2012).

The selection of the best primer pair was conducted in two phases, except for the *Anser* primers, which were selected unaltered from a previous study (Honka et al., 2018). In the first phase, we tested the primers developed or modified in this study by amplifying modern DNA of the focal species, either *B. leucopsis* or *C. cygnus*. These samples were moulted feathers, from which DNA was extracted from the calamus and blood clot as in Honka et al. (2022).

PCR was performed in a temperature gradient in order to experimentally determine the best annealing temperatures for the primer pairs. The PCR conditions were as follows: 1 × Phusion HF‐buffer (Thermo Fisher Scientific), 0.20 mM dNTPs, 0.5 μM of each primer, 0.02 U/μL Phusion High‐Fidelity DNA Polymerase (Thermo Fisher Scientific) and 1 μL of extracted DNA. The thermal profile consisted of 98°C for 4 min, followed by 40 cycles of 98°C for 30 s, 53–63°C (temperature gradient) for 30 s and 72°C for 40 s, with a final extension of 72°C for 7 min. The best annealing temperatures were determined to be 63°C for *B. leucopsis* and 57°C for *C. cygnus*.

### 2.5 Testing primers for environmental DNA

All primer pairs produced PCR products of the correct size in the feather samples of the barnacle goose and the whooper swan (*Anser* primers tested previously). Next, we separately amplified the eDNA samples using two different primer pairs for the bean goose, three different primer pairs for *Branta* and three different primer pairs for the whooper swan (altogether 8 PCR reactions) (Table 2). For *Branta*, we observed that with primer pair BrCytB2‐F/R, the PCR product only sequenced in one direction, and we redesigned the reverse primer. We also tested the primer pair BrCytB2‐F/R2 (altogether 9 PCR reactions). We used the ‘Rescue‐PCR’ protocol, designed to reduce PCR inhibition by increasing the amount of reagents by 25% (Johnson & Kemp, 2017), and as no amplification was observed using a standard PCR protocol (manufacturer's recommendation, no increase in reagent amounts).

PCR reactions were performed in 25 μL reaction volumes with the following conditions: 1.25 × Phusion HF‐buffer (Thermo Fisher Scientific), 0.25 mM dNTPs, 0.5 μM of F‐ and R‐primer, 3.1 mM MgCl_2_, 1 mg/mL BSA (Bovine serum albumin), 0.03 U/μL Phusion High‐Fidelity DNA Polymerase (Thermo Fisher Scientific) and 2 μL of extracted DNA. The thermal profile consisted of 98°C for 4 min, followed by 55 cycles of 98°C for 30 s, 57°C for *Anser* sp., 63°C for *Branta* sp., and 57°C for *Cygnus* sp. for 30 s, and 72°C for 40 s with a final extension of 72°C for 7 min. Throughout all PCRs, we run negative controls including water instead of DNA template. The PCR products were checked on a 2% agarose gel with 0.5 × TBE and 3 μL of Midori Green Advance DNA stain (Nippon Genetics), and ran for 50 min on 115 volts.

**FIGURE A5:**
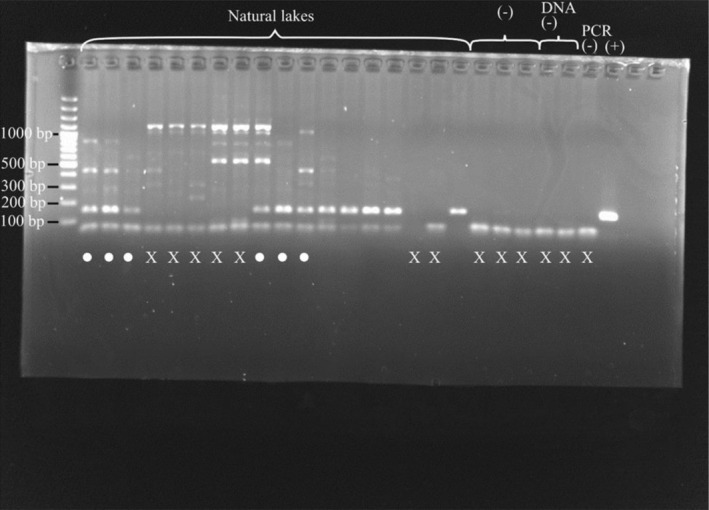
Agarose gel image of a PCR for environmental samples using *Anser*‐specific primers AdCR2‐F and AdCR2‐R. The first well is the ladder (GeneRuler 100 bp Plus DNA Ladder, ThermoFisher Scientific), wells 2–19 are eDNA samples from lakes, wells 20–22 are field negative controls (sterile water), wells 23–24 are negative controls from DNA extraction, well 25 is PCR negative control and well 26 is a positive control, which is bean goose DNA extracted from a muscle tissue sample. The white dots indicate samples that were gel extracted and white X indicates failed samples (no PCR product of correct size, only primer dimers).

**FIGURE A6:**
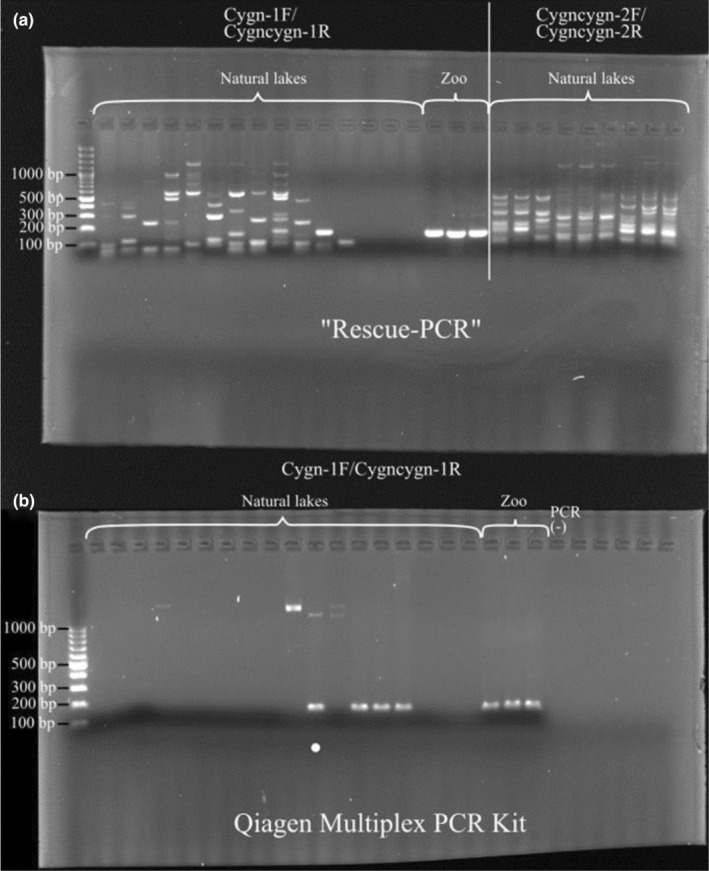
Agarose gel image of a PCR of environmental samples using *Cygnus*‐specific primers Cygn‐1F and Cygncygn‐1R. (a) eDNA samples with ‘Rescue‐PCR’ protocol. The first well is the ladder (GeneRuler 100 bp Plus DNA Ladder, ThermoFisher Scientific), wells 2–16 are eDNA samples from lakes, wells 17–19 are zoo pond samples and wells 20–27 are natural lakes with primer pair Cygncygn‐2F and Cygncygn‐2R (omitted from final analyses). (b) eDNA samples with Qiagen Multiplex PCR Kit. The first well is the ladder (GeneRuler 100 bp DNA Ladder, ThermoFisher Scientific), wells 2–19 are eDNA samples from lakes, wells 20–22 are zoo pond samples and well 23 is PCR negative control. The white dot indicates a sample that was gel extracted.

The primer pair AdCR1‐F and AdCR1‐R did not produce a PCR product of the correct size and was thus found to be unsuitable for eDNA (results not shown). However, the primer pair AdCR2‐F and AdCR2‐R produced PCR bands of the correct size in 11 samples for the taiga bean goose (Figure A5 in Appendix). Six of these samples had non‐specific PCR products co‐amplifying with the PCR fragment of correct size (Figure A5 in Appendix). The PCR products of the correct size were cut from the gel and extracted using GeneJET Gel Extraction Kit (Thermo Fisher Scientific) according to the manufacturer's instructions. The PCR products were purified with Fast‐AP (Thermo Fisher Scientific) and ExoI (Thermo Fisher Scientific) enzymatic purification. All samples (PCR products of the correct size and gel‐extracted) were sequenced to both directions with BigDye Terminator v.3.1 (Applied Biosystems) chemistry using the PCR primers and the reactions were run on an ABI 3730 (Applied Biosystems). The ‘Rescue‐PCR’ protocol produced primer dimers (PCR product <100 bp) in almost all samples and negative controls with *Anser* primers (Figure A5 in Appendix), but the correctly sized products were identifiable. PCRs for the genus *Branta* and whooper swan produced so many non‐specific PCR products that it was difficult to estimate the correctly sized PCR products (Figure A6 in Appendix) and thus the performance of the primers.

### 2.6 Optimisation of PCR protocol

Due to the poor performance of the ‘Rescue‐PCR’ with the genus *Branta* and *Cygnus*, we tested a Hot‐start Phusion Hot Start II DNA Polymerase (Thermo Fisher Scientific) and the Qiagen Multiplex PCR Kit (Qiagen) which contains a modified Hot‐start *Taq*‐enzyme. Hot‐start enzymes are designed to minimise the amounts of non‐specific PCR products as the polymerase is not active during the reaction mixture setup and is only activated when heated to over 90°C. This prevents the formation of non‐target amplification.

To test the performance of different PCR protocols, we used primers AdCR2‐F and ‐R (*Anser*‐genus) and selected one sample (EB12) which produced a single PCR product, one sample (EB1) which was gel extracted due to additional non‐specific fragments and one sample (EB4) which failed previously with the ‘Rescue‐PCR’ protocol using the non‐Hot‐start Phusion enzyme. In addition, we tested the effect of diluting the DNA extracts (undiluted, 5, and 10 ng/μL) with the different PCR protocols, as diluting the DNA extracts could also dilute the PCR inhibitors potentially present in our samples.

The first tested PCR protocol was performed in 25 μL reaction volume with the following PCR conditions: 1 × Phusion HF‐buffer (Thermo Fisher Scientific), 0.20 mM dNTPs, 0.5 μM of each primer, 2.5 mM MgCl_2_, 1 mg/mL BSA (Bovine serum albumin), 0.02 U/μL Phusion Hot Start II DNA Polymerase (Thermo Fisher Scientific) and 2 μL of extracted DNA. The thermal profile consisted of 98°C for 4 min, followed by 55 cycles of 98°C for 30 s, 63°C for 30 s and 72°C for 40 s with a final extension of 72°C for 7 min. The annealing temperature was raised to 63°C to increase the specificity of the primer‐template priming.

The second tested protocol was the same as the first one, but reagents were increased by 25% as in ‘Rescue‐PCR’ (Johnson & Kemp, 2017) with the exception of primers that had heavy primer dimers visible on the gel images. The PCR was performed in 25 μL reaction volume with 1.25 × Phusion HF‐buffer (Thermo Fisher Scientific), 0.25 mM dNTPs, 0.5 μM of F‐ and R‐primer, 3.1 mM MgCl_2_, 1 mg/mL BSA (Bovine serum albumin), 0.03 U/μL Phusion Hot Start II DNA Polymerase (Thermo Fisher Scientific) and 2 μL of extracted DNA. The thermal profile consisted of 98°C for 4 min, followed by 55 cycles of 98°C for 30 s, 63°C for 30 s and 72°C for 40 s, with a final extension of 72°C for 7 min.

The third tested protocol was a touchdown PCR. The touchdown protocols start from a higher than optimal annealing temperature ensuring very specific primer‐template binding and incrementally lowering the annealing temperature to optimal to ensure high yield. The specific PCR products produced in the first cycles act as templates for later cycles, theoretically ensuring that unspecific products are not produced, or are produced in such low amounts that the correct product overrules. PCR reactions were as in the first protocol, except the reactions were amplified in 15 μL reaction volumes with 1.2 μL of DNA, and the cycling conditions were as follows: 98°C for 4 min, followed by 2 cycles of 98°C for 30 s, 68°C for 30 s, and 72°C for 40 s. This was followed by lowering the annealing temperature incrementally by two degrees every two cycles until 58°C was reached, after which 43 cycles of 98°C for 30 s, 57°C for 30 s and 72°C for 40 s were repeated with a final extension of 72°C for 7 min.

The fourth tested protocol was similar to the first, with the exception of performing the PCR reactions in 15 μL reaction volumes with 1.2 μL of DNA template and reducing the PCR cycling times as follows: 98°C for 4 min, followed by 45 cycles of 98°C for 1 s, 63°C for 15 s and 72°C for 15 s, with a final extension of 72°C for 1 min as in ‘Fast PCR’ (Sullivan et al., 2006). ‘Fast PCR’ could reduce the amplification of unspecific products because the non‐specific products are much larger in size than the targeted DNA, and thus, using very short annealing and extension times theoretically prevents the polymerase from amplifying the longer fragments.

The fifth tested PCR protocol was performed in 10 μL volumes with 1 × QIAGEN Multiplex PCR Master Mix (Qiagen) containing HotStarTaq DNA polymerase, 0.2 μM of F‐ and R‐primers and RNAse‐free water. The PCR‐cycling conditions were as follows: 95°C for 4 min, followed by 45 cycles of 94°C for 30 s, 59°C for 90 s and 72°C for 90 s with a final extension of 72°C for 10 min.

**FIGURE A7:**
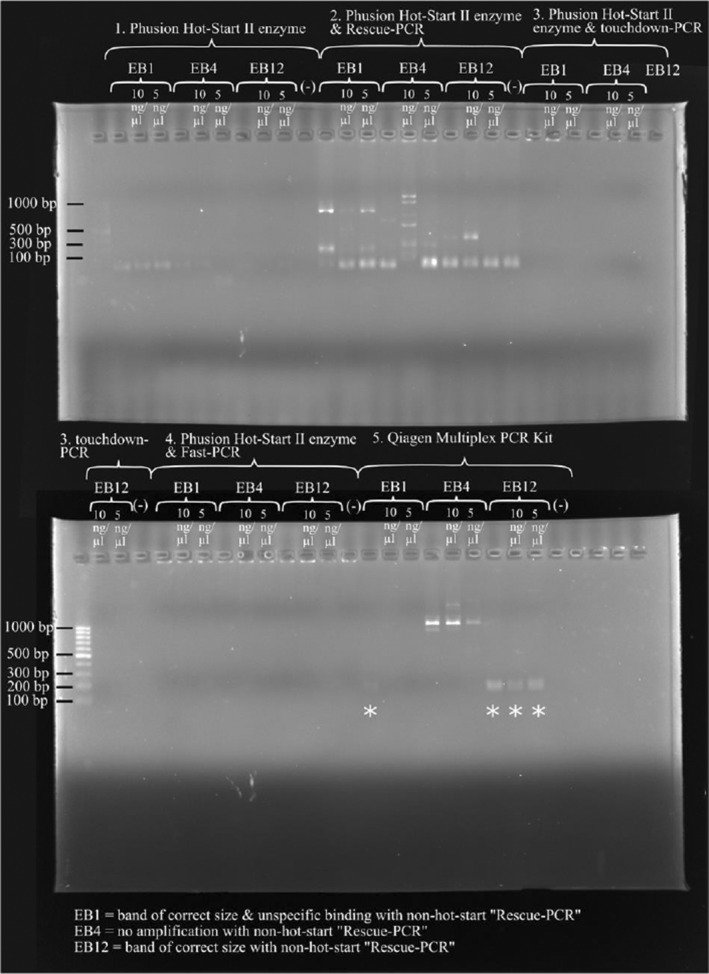
Five different tested PCR protocols run on a 2% agarose gel. Each protocol was tested with three samples (EB1, EB4 and EB12) in three different concentrations: undiluted, 10 and 5 ng/μL. The first wells in the gels contain the ladder (GeneRuler 100 bp DNA Ladder, ThermoFisher Scientific), Asterisk (*) means samples which yielded easily interpretable PCR band of a correct size. Note that the band in sample EB1 in protocol 5 (Qiagen Multiplex PCR Kit) is very faint, and thus barely visible in the image, but was visible when the gel was lighted in a UV‐table. Negative controls are marked as (−).

The only PCR protocols which worked in our test were ‘Rescue‐PCR’ with Phusion Hot‐start enzyme and the Qiagen Multiplex PCR Kit. However, the ‘Rescue‐PCR’ with Phusion Hot‐start enzyme produced primer dimers and non‐specific PCR products (Figure A7 in Appendix), making the interpretation of correctly sized bands very difficult (no improvement compared to non‐Hotstart Phusion enzyme), while the Qiagen Multiplex PCR Kit produced no primer dimers or unspecific binding, except only in the sample which failed to yield bean goose DNA. The unspecific binding in this sample was probably because it did not contain any bean goose DNA (no primer annealing sequence), and thus non‐target sequences were amplified. The Qiagen Multiplex PCR Kit has the additional benefit that no PCR additives (BSA) were needed, and as the product is a master mix, the contamination probability is lower as fewer tubes are needed to be opened when preparing the PCR mix.

### 2.7 PCR for eDNA using the Qiagen multiplex PCR kit

We performed PCR in 10 μL volumes with 1 × QIAGEN Multiplex PCR Master Mix, 0.2 μM of F‐ and R‐primers, and RNAse‐free water for *Branta* species and whooper swan. The PCR‐cycling conditions were following: 95°C for 15 min, followed by 45 cycles of 94°C for 30 s, 63°C for *Branta* sp. and 57°C for *Cygnus* sp. for 90 s, and 72°C for 90 s with a final extension of 72°C for 10 min. The PCR products were run on a 2% agarose gel and extracted from the gel using the GeneJET Gel Extraction Kit if needed. The best results were obtained with primers BrCytB2‐F and BrCytB2‐R2 for the genus *Branta*, and Cygn‐1F and Cygncygn‐1R for the genus *Cygnus* (Table 2).

We determined the limit of detection (LOD) by quantifying the concentration of DNA extracted from a taiga bean goose muscle tissue sample (16.4 ng/μL) using PicoGreen dsDNA Assay Kit (Thermo Fisher Scientific). We then diluted this DNA to 10 ng/μL and created a 1:10 dilution series from 10 to 0.000001 ng/μL. The PCR protocol was performed as above with the Qiagen Multiplex Kit and the PCR products were run on a 2% agarose gel.

The successfully amplified PCR products were purified with Fast‐AP (Thermo Fisher Scientific) and ExoI (Thermo Fisher Scientific) enzymatic purification. Subsequently, the samples were sequenced in both directions with BigDye Terminator v.3.1 (Applied Biosystems) chemistry with the PCR primers and the reactions were run on an ABI 3730 (Applied Biosystems).

### 2.8 Sequence analysis

We manually edited the sequences using the program CodonCode Aligner v.4.0.4. (CodonCode Corporation). Some sites exhibited unresolved nucleotides, that is, several nucleotides existed at a single site. The presence of these sites indicates either the presence of multiple haplotypes of a single species (i.e. at least two individuals with different haplotypes) or the presence of multiple species. To phase the haplotype data, we used DnaSP v5. (Librado & Rozas, 2009) with our custom databases (see below for each genus). This allowed us to accurately distinguish between different haplotypes and species.

We used the program BioEdit 7.2.5 (Hall, 1999) to align the *Anser* sequences from eDNA samples with GenBank sequences from all *Anser* species (see Table A1 in Appendix). Similarly, the *Branta* sequences from eDNA samples were aligned against the GenBank sequences of species in the genus *Branta*, and the swan sequences from eDNA samples were aligned with GenBank sequences from the *Cygnus* species (see Table A1 in Appendix). We used the program PopART (Leigh & Bryant, 2015) to construct median‐joining networks (Bandelt et al., 1999) separately for these three alignments. This approach allowed us to verify that the correct species was amplified by examining the genetic relationships within the obtained sequences.

## 3 RESULTS

### 3.1 Primer design and PCR optimisations

We tested several primer pairs first with a feather sample of the barnacle goose and the whooper swan. We found that newly developed primer pairs for *Branta* detection and the modified primer pairs for *Cygnus* detection all amplified the target species. Next, we tested all primer pairs with eDNA samples and found that not all the primer pairs were suitable for eDNA. The best‐performing primer pair for *Branta* eDNA was BrCytB2‐F/R2 and Cygn1‐F/Cygngygn1‐R for *Cygnus*. For *Anser*, we tested two primer pairs from the literature, and the primer pair AdCR2‐F/R was found to be suitable for eDNA. We also found that Qiagen's Multiplex PCR kit performed the best with eDNA samples after testing two Hotstart polymerase enzymes and several different PCR protocols.

We successfully detected amplification with taiga bean goose tissue DNA diluted to 0.00001 ng/μL when visualised on agarose gel, and a lack of amplification with the more diluted sample (0.000001 ng/μL). Therefore, we established the limit of detection to be 0.00001 ng/μL for the taiga bean goose tissue sample.

### 3.2 eDNA presence/absence data

**FIGURE 2:**
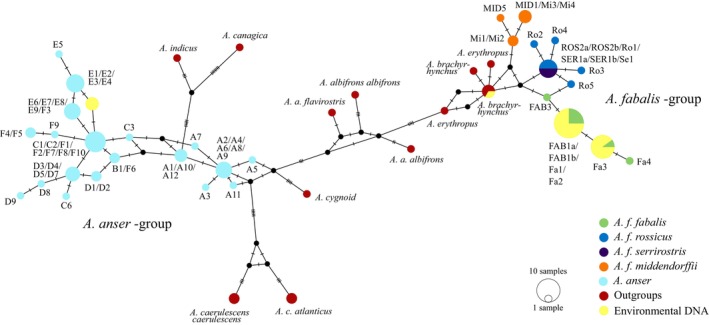
A median‐joining haplotype network for the different bean goose subspecies (*Anser fabalis fabalis*, *A. f. rossicus*, *A. f. serrirostris* and *A. f. middendorffii*), different greylag goose (*A. anser*) haplotypes and all *Anser* species as outgroups. *Anser* species can be separated from each other using this short mitochondrial DNA region (102 bp). The environmental DNA (eDNA) samples (including resolved haplotypes) are shown in a yellow colour group with the taiga bean goose sequences except for one haplotype which groups with the pink‐footed goose (*A. brachyrhynchus*). The sizes of the circles are proportional to the frequency of each haplotype and tick marks across branches indicate the number of mutational differences. Forward slashes between haplotype names denote identical haplotypes based on the sequenced fragment but differ based on the whole control region sequence of the bean geese.

Based on the sequencing results, we detected taiga bean goose DNA in five of the six natural lakes where the species had been visually observed (Table 1, Figure 2), in addition to the zoo pond.

**FIGURE 3:**
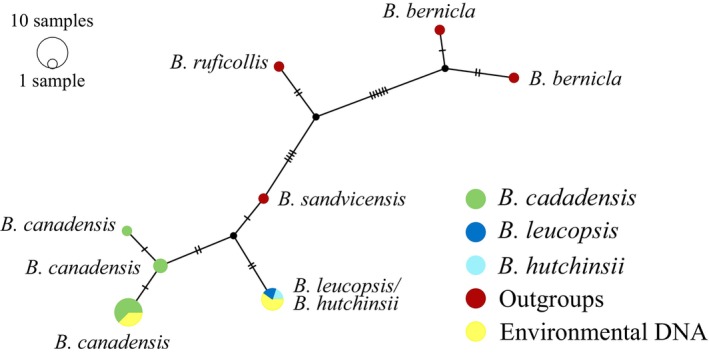
A median‐joining network for Canada goose (*Branta canadensis*), barnacle goose (*B. leucopsis*), cackling goose (*B. hutchinsii*) and other *Branta* species as outgroups for 169 bp of *cytochrome b* sequence. The environmental DNA (eDNA) samples are shown in yellow colour. After phasing the samples grouped with Canada goose and barnacle/cackling groups due to sequence similarity of barnacle and cackling goose. Overlap in the ranges of these species is limited but should be taken into account in further studies. The sizes of the circles are proportional to the frequency of each haplotype and tick marks across branches indicate the number of mutational differences.

No DNA of *Branta* species was detected from the six natural lakes, which was expected since no sightings of the genus *Branta* were made in these lakes (which are not in their breeding range). It should be noted that the cackling goose and barnacle goose DNA were indistinguishable in the studied DNA region, but cackling goose does not exist in Finland. We detected both Canada goose and barnacle goose/cackling goose DNA from all of the zoo replicates (Figure 3, Table 1), but the cackling goose was not present in the zoo pond; thus the other species was barnacle goose.

**FIGURE 4:**
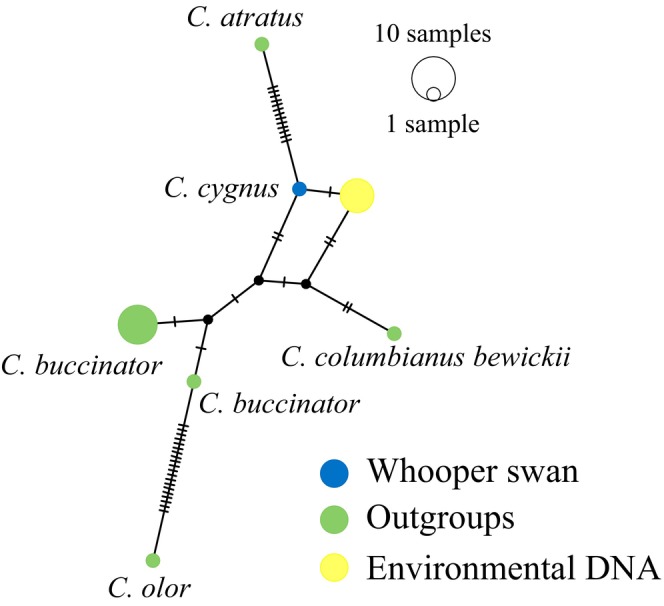
A median‐joining haplotype network for the whooper swan (*Cygnus cygnus*) and other *Cygnus* species as outgroups for 121 bp of the mitochondrial control region. The environmental DNA (eDNA) samples shown in yellow colour share the same haplotype which differs by one nucleotide from the whooper swan GenBank sequence (accession number: NC_027095). The sizes of the circles are proportional to the frequency of each haplotype and tick marks across branches indicate the number of mutational differences.

Whooper swan DNA was detected from three of the six natural lakes and in the zoo sample (Table 1, Figure 4). Most of the detected whooper swan DNA was amplified with primer pair Cygn‐1F/Cygncygn‐1R, but in the zoo samples, the sequence quality was low.

### 3.3 Sequencing results

In three natural lakes, all three replicate samples contained taiga bean goose DNA, while in two sites only one of the replicate samples contained taiga bean goose DNA (Table 1). One of the replicates (EB18) contained both taiga bean goose and pink‐footed goose DNA after phasing the haplotypes. However, it should be noted that pink‐footed goose‐type mtDNA has been found in bean geese breeding in Finland (Honka et al., 2022), and the presence of this haplotype does not necessarily indicate the presence of pink‐footed goose, as it might also result from mitochondrial introgression. Most of the natural lakes contained two bean goose haplotypes (FAB1a/FAB1b/Fa1/Fa2 and Fa3) after phasing (Table 1) indicating the presence of at least two bean goose individuals with different mtDNA haplotypes. The slashes between haplotype names denote identical haplotypes in the studied fragment, but differing haplotypes when analysing the whole control region.

Even though the zoo pond housed three different *Anser* species, we were able to detect the DNA of the taiga bean goose there, in addition to greylag goose DNA after phasing, but not the lesser white‐fronted goose (Figure 2, Table 1). However, the variable sites in the eastern haplotypes of lesser white‐fronted goose could be masked by the variation present in the bean goose and the greylag goose when chromatograms were inspected by eye. However, in natural environments, the presence of all three species together in breeding sites is highly unlikely due to the different breeding habitats of the greylag and lesser white‐fronted goose.

## 4 DISCUSSION

We developed primers for an environmental DNA‐based detection method for large waterfowl breeding in the Northern Hemisphere, the true geese (*Anser* and *Branta*) and swans (*Cygnus*). This method could be used in population monitoring or mapping the distribution of endangered or poorly known populations. Specifically, we focused on the taiga bean goose, visually confirmed in all the sampled lakes, as well as Canada goose, barnacle goose and whooper swan. We used genus‐level primers and confirmed the occurrence of single species using Sanger sequencing, which is vital in the case that primers amplify several species, as in here. We found no suitable regions for primers to develop species‐specific primers, which inhibited the use of, for example, quantitative PCR. We also tested different PCR protocols, polymerases and eDNA dilutions, and the Qiagen Multiplex PCR Kit was found to perform the best with eDNA samples. Additionally, undiluted eDNA showed the best PCR amplification and thus we do not recommend diluting eDNA extracts.

The taiga bean goose population has been declining since the 1990s, and is of management concern, especially given its status as a hunted species. The developed eDNA assay holds potential applications in mapping breeding distribution, monitoring local populations and studying the utilisation of breeding/brooding sites over different years. The taiga bean goose DNA failed to amplify in one of the six natural lakes where the species was visually observed. There could be various reasons for this. We do not know how much time the individuals had spent in each studied lake. Therefore, it remains uncertain if the lake was used regularly by the geese, for example, for feeding, or if it was only briefly visited potentially resulting in a lack of amplifiable DNA. At the time of our sampling, observations were made from broods that were moving widely and potentially changing roosting sites. Additionally, empirical evidence has shown that in lakes, eDNA is not evenly distributed, its dispersal is spatially limited and the dispersal distances vary between different aquatic species (Brys et al., 2021). Therefore, it is also possible that by chance we did not sample the part of the lake where the bean goose DNA resided in the water.

Moreover, lake chemistry could be a contributing factor to the PCR failure, as factors such as pH, CO_2_ or O_2_, in addition to water temperature, turbidity, acidity, salinity and UV exposure can influence eDNA release and persistence (as reviewed in Harrison et al., 2019 and Stewart, 2019). In the present study, the water properties were not measured in the sampled lakes. In a study involving Gouldian finches, it was observed that if finches were not present 72 h prior to eDNA sampling, the eDNA detection yielded negative results (Day et al., 2019). Therefore, if the geese had visited the lake several days before sampling, it is plausible that the eDNA had decayed. The primers used for detecting the taiga bean goose can also be used to detect other bean goose subspecies, such as the tundra bean goose (*A. f. rossicus*), due to their sequence similarity. The tundra bean goose breeds in very low numbers in the northernmost Finnish Lapland and its breeding range is poorly known in this region. While our primers theoretically have the potential to detect all *Anser* species, empirical testing for each species is essential to avoid false negatives. For example, eDNA could be used to locate lakes occupied by the critically endangered Fennoscandian lesser white‐fronted goose (*A. erythropus*) in their former breeding grounds in Lapland of Finland, Sweden and Norway.

The detection of *Branta* species was not expected in the sampled lakes, given that these species primarily inhabit southern Finland or the coastline (Valkama et al., 2011). Therefore, the samples from Northern Finland were not expected to contain these species or, if present, they would be very rare. The Canada goose is an introduced North American species, which has established a breeding population of 7000–8000 pairs in Finland from birds introduced in the 1960s (Valkama et al., 2011). The barnacle goose, which mainly breeds in Novaja Zemlya, Greenland and Svalbard, began breeding in Finland in the 1980s (Valkama et al., 2011) and increased as a breeding bird in recent decades to a population of around 26,900 individuals in Finland (BirdLife Suomi ry, 2023a). It is noteworthy that the North American cackling goose shares an identical sequence in the studied DNA fragment with the European breeding barnacle goose. The cackling goose in Finland is extremely rare, with only two individuals observed in the wild and few observed as zoo or farm escapees (after 1949; BirdLife Suomi ry, 2023b). These two species generally do not co‐occur, except in Western Europe in their wintering ranges where the cackling goose is a rare winter visitor. More primer development is needed for regions in which these two species can co‐occur, as well as further testing within the range of Canada goose and barnacle goose.

The whooper swan is a common breeding bird throughout Finland (Valkama et al., 2011), making its presence expected in any of the lakes. This species was confirmed in a lake in which the whooper swan was seen during the eDNA sampling. The developed primers could be also suitable for detecting other northern swan species, such as the tundra swan (*C. columbianus*) and the trumpeter swan (*C. buccinator*), although further testing is needed for these species. Additionally, the primers could be further modified to suit the detection of mute swan (*C. olor*). Observations have shown that the whooper swans act aggressively toward bean geese, and it has even been proposed that an increased whooper swan population could have contributed to the decline of the taiga bean goose population (Kampe‐Persson et al., 2005). However, evidence for this is lacking. With the help of the eDNA method presented here and a larger sample size, it could be determined whether whooper swans and bean geese share lakes. Increasing numbers of whooper swans or Canada geese have not negatively affected populations of smaller waterbirds, indicating no resource competition among them (Holopainen et al., 2022), but such studies have not been performed among large waterfowl.

The results obtained here demonstrate the suitability of the eDNA‐based methods for waterfowl detection. However, the success of this method relies on meticulous primer design and the selection of an appropriate PCR protocol. For instance, Sanger sequencing is the preferable downstream method when sequence divergence between the studied species and the co‐occurring species is low, while quantitative PCR is more suitable when sequence divergence allows for the development of species‐specific primers. For example, Neice and McRae (2021) found that the sensitivity of the black rail eDNA qPCR was much higher than that of PCR combined with Sanger sequencing, with a hundredfold difference in detection limit. Our results align with findings from other single‐species eDNA detections in birds (Day et al., 2019; Feist et al., 2022; Neice & McRae, 2021), which are currently limited to two marshland birds and two land birds.

In the future, the monitoring of goose and swan populations in their breeding grounds could be conducted using eDNA and sequencing of PCR‐amplified products. The method employed in this study eliminates the need for immediate sample freezing after collection. Instead, samples can be stored in ambient temperatures, facilitating sampling in remote areas with no access to freezers. eDNA could significantly improve the detection of elusive goose species in their Arctic breeding areas which are difficult to reach and where visual field monitoring is challenging. Several goose species are of management concern and eDNA‐based detection methods could be beneficial in mapping the distributions of highly endangered populations, monitoring declining species or studying the co‐occurrence of several waterfowl taxa. Metabarcoding using the primers developed here would be beneficial in situations in which multiple goose species are expected to occur in the same lake, such as in staging and wintering areas. This approach allows for the simultaneous detection and identification of multiple species from environmental DNA samples, providing a comprehensive understanding of the species composition within a given habitat.

## AUTHOR CONTRIBUTIONS

**Johanna Honka:** Conceptualization (equal); formal analysis (lead); investigation (lead); methodology (lead); visualization (lead); writing – original draft (lead); writing – review and editing (equal). **Laura Kvist:** Conceptualization (equal); formal analysis (supporting); investigation (supporting); methodology (supporting); supervision (equal); writing – original draft (supporting); writing – review and editing (equal). **Suvi Olli:** Formal analysis (supporting); investigation (equal); writing – original draft (supporting); writing – review and editing (equal). **Toni Laaksonen:** Investigation (equal); resources (equal); writing – original draft (supporting); writing – review and editing (equal). **Jouni Aspi:** Conceptualization (equal); formal analysis (supporting); investigation (supporting); methodology (supporting); supervision (equal); writing – original draft (supporting); writing – review and editing (equal).

## CONFLICT OF INTEREST STATEMENT

We declare no conflict of interest.

## Data Availability

The sequences of the new haplotype for the whooper swan and the sequence of the zoo greylag goose after phasing are provided in the Appendix as Text A1. The sequence alignments are available from the corresponding author upon reasonable request.

## Appendix

**Text A1**

Sequence of the eDNA of the Finnish whooper swans:

TTATAATCCCCATACATATAACTATGGTCCCAGTAATACGCGTTACGCACGGACTAGCCCACAAGCAAGTACTAAACCCATAACATGCAAACGGACATCAAACCCTAACAGCACTTCCCT.

Sequence of the eDNA of the zoo greylag geese after phasing:

CCCACAACACCCAACACAACTCTAGCTCAAGCACACAACAAGGCCCCATTTTAATGAATGCTCACAGGACATACCCTAACAACAATCCTCTACCACATATCT.

**TABLE A1:** Custom sequences database species, GenBank accession numbers and references.

Species common name	Species name	GenBank accession number	References
*Anser*‐database			
Bean goose	*Anser fabalis*	EU186805–EU186812	Ruokonen et al. (2008)
Bean goose	*Anser fabalis*	AF159951	Ruokonen et al. (2000)
Bean goose	*Anser fabalis*	MH491806–MH491819	Honka et al. (2017)
Pink‐footed goose	*Anser brachyrhynchus*	AF159952–AF159954	Ruokonen et al. (2000)
Lesser white‐fronted goose	*Anser erythropus*	AF159955–AF159956	Ruokonen et al. (2000)
Greater white‐fronted goose	*Anser albifrons*	AF159957–AF159959	Ruokonen et al. (2000)
Swan goose	*Anser cygnoid*	AY072581	Paxinos et al. (2002)
Greylag goose	*Anser anser*	AF159961–AF159963	Ruokonen et al. (2000)
Bar‐headed goose	*Anser indicus*	KM455570	Mu and Yu (2014)
Snow goose	*Anser caerulescens*	FJ905228, FJ905257 and FJ905285	Humphries et al. (2009)
Ross's goose	*Anser rossii*	AY072582	Paxinos et al. (2002)
Emperor goose	*Anser canagica*	AY072583	Paxinos et al. (2002)
Numt		AF159970	Ruokonen et al. (2000)
*Branta*‐database			
Canada goose	*Branta canadensis*	EU585629	Gonzalez et al. (2009)
Canada goose	*Branta canadensis*	FJ423757	Spicer and Banbury (2008)
Canada goose	*Branta canadensis*	MH676096–MH676101	Anaya et al. (2018)
Cackling goose	*Branta hutchinsii*	MH676095	Anaya et al. (2018)
Barnacle goose	*Branta leucopsis*	EU585630	Gonzalez et al. (2009)
Red‐breasted goose	*Branta ruficollis*	EU585631	Gonzalez et al. (2009)
Brent goose	*Branta bernicla*	EU585628	Gonzalez et al. (2009)
Brent goose	*Branta bernicla*	HM063580	Bulgarella et al. (2010)
Hawaiian goose	*Branta sandvicensis*	EU585632	Gonzalez et al. (2009)
*Cygnus*‐database			
Whooper swan	*Cygnus cygnus*	NC_027095	Park et al. (2016)
Mute swan	*Cygnus olor*	NC_027096	Park et al. (2016)
Tundra swan	*Cygnus columbianus*	NC_017604	Lee et al. (2012)
Trumpeter swan	*Cygnus buccinator*	EF165350–EF165358	Oyler‐McCance et al. (2007)
Black swan	*Cygnus atratus*	AY112978	Donne‐Goussé et al. (2002)
